# Subcellular Location of PKA Controls Striatal Plasticity: Stochastic Simulations in Spiny Dendrites

**DOI:** 10.1371/journal.pcbi.1002383

**Published:** 2012-02-09

**Authors:** Rodrigo F. Oliveira, MyungSook Kim, Kim T. Blackwell

**Affiliations:** The Krasnow Institute for Advanced Study, George Mason University, Fairfax, Virginia, United States of America; Indiana University, United States of America

## Abstract

Dopamine release in the striatum has been implicated in various forms of reward dependent learning. Dopamine leads to production of cAMP and activation of protein kinase A (PKA), which are involved in striatal synaptic plasticity and learning. PKA and its protein targets are not diffusely located throughout the neuron, but are confined to various subcellular compartments by anchoring molecules such as A-Kinase Anchoring Proteins (AKAPs). Experiments have shown that blocking the interaction of PKA with AKAPs disrupts its subcellular location and prevents LTP in the hippocampus and striatum; however, these experiments have not revealed whether the critical function of anchoring is to locate PKA near the cAMP that activates it or near its targets, such as AMPA receptors located in the post-synaptic density. We have developed a large scale stochastic reaction-diffusion model of signaling pathways in a medium spiny projection neuron dendrite with spines, based on published biochemical measurements, to investigate this question and to evaluate whether dopamine signaling exhibits spatial specificity post-synaptically. The model was stimulated with dopamine pulses mimicking those recorded in response to reward. Simulations show that PKA colocalization with adenylate cyclase, either in the spine head or in the dendrite, leads to greater phosphorylation of DARPP-32 Thr34 and AMPA receptor GluA1 Ser845 than when PKA is anchored away from adenylate cyclase. Simulations further demonstrate that though cAMP exhibits a strong spatial gradient, diffusible DARPP-32 facilitates the spread of PKA activity, suggesting that additional inactivation mechanisms are required to produce spatial specificity of PKA activity.

## Author Summary

The striatum is a part of the basal ganglia which plays a role in addiction and reward learning. Its importance is underscored by pathologies such as Parkinson's disease and Huntington's disease in which degeneration of the dopamine inputs to the striatum or degeneration of neurons in the striatum, respectively, produces motor dysfunction. Dopamine in the striatum activates cascades of signaling molecules, ultimately producing an activity dependent change in the strength of connections between neurons. However, the dispersive movement of signaling molecules seems incompatible with the strengthening of specific subsets of connections, which is required for formation of distinct memories. Anchoring proteins, which restrict molecules to particular compartments within the neuron, are proposed to achieve specificity. We develop a reaction-diffusion model of dopamine activated signaling pathways to explore mechanisms whereby anchoring proteins can produce specificity. We use an efficient Monte-Carlo simulator to implement the cascades of signaling molecules in a neuronal dendrite with multiple dendritic spines. Simulations demonstrate that spatial specificity requires both anchoring proteins and inactivation mechanisms that limit the diffusion of signaling molecules.

## Introduction

The striatum is the primary input nucleus of the basal ganglia, a set of forebrain nuclei implicated in addiction [1], motor control [2], and reinforcement learning [3], [4]. The function of the striatum is attributed to integration of glutamatergic inputs from cortex and thalamus [5] with dopaminergic afferents from midbrain [6]. In particular, dopamine signaling plays a preeminent role in striatal dependent learning [2] and in synaptic plasticity of the medium spiny projection neurons (MSPN) [7], [8]. The importance of dopamine is underscored by Parkinson's disease [9], which is caused by degeneration of dopamine producing neurons. Therefore understanding intracellular signaling produced by dopamine receptor activation is paramount for understanding the role of the striatum in learning and motor function.

Dopamine binding to the D1 receptor leads to production of cAMP which activates protein kinase A (PKA). PKA phosphorylation of DARPP-32 (Dopamine- and cAMP-regulated phosphoprotein with molecular weight 32 kD) and Ser845 of the AMPA receptor GluA1 subunit have been implicated in learning [10], addiction [1] and synaptic plasticity [7], [11]. The spatial specificity of synaptic plasticity [12]–[14] seems incompatible with diffusion of signaling molecules such as cAMP; thus, mechanisms are required to produce local activation, i.e. microdomains.

Creation of microdomains depends on several factors such as diffusional barriers, embodied by dendritic spines [15], [16], or inactivation mechanisms [17], exemplified by phosphodiesterases [18]. Development of microdomains is facilitated by multi-protein complexes, such as produced by the A-kinase-anchoring-protein AKAP5 [19], which is highly expressed in the striatum [20], [21]. AKAPs bind not only PKA but diverse molecules such as adenylate cyclase [22], and sodium channels [23]. These multi-protein complexes promote efficient PKA activation and keep its activity spatially constrained [24]. Consistent with this role, blocking anchoring of PKA to the AKAP disrupts hippocampal late-phase long term potentiation (LTP) [25], sodium channel phosphorylation [23] and striatal LTP [26]. Under conditions of synaptic activation, it is unclear whether the crucial role of AKAPs is to anchor PKA near adenylate cyclase, the cAMP source, or close to the target substrate.

Several computational modeling studies [27]–[29] have investigated the control of PKA activity and DARPP-32 phosphorylation by dopamine receptor activation, but the formation of microdomains and the role played by the subcellular location of molecule species remains largely unexplored. To resolve these important issues, we investigate the role of PKA anchoring using a spatial, Monte Carlo model of dopamine activated post-synaptic signaling pathways in a MSPN dendrite with multiple spines. Simulations demonstrate that colocalization of PKA with the source of cAMP is more important than colocalization with target molecules, and demonstrate the role of inactivation mechanisms for the generation of microdomains.

## Methods

### Signaling Pathways

**Figure 1:**
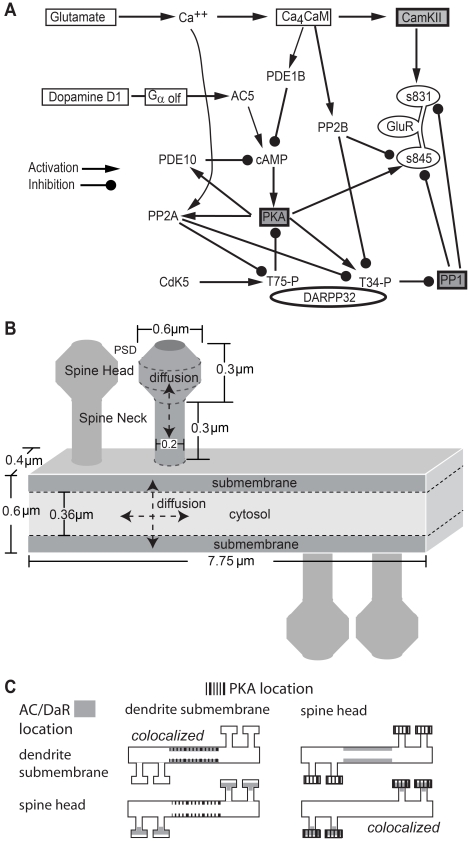
Model of striatal medium spiny projection neuron dendrite with spines. A. Diagram of biochemical signaling pathways. Each arrow is modeled with one or more bimolecular or enzyme reactions. See text and Table 1 for details. B. Morphology of dendrite with four spines, and location of calcium influx in the model. Subvolumes of height 0.12 µm adjacent to the top and bottom surface of the dendrite are considered submembrane subvolumes. Other dendritic subvolumes are part of the cytosol. Diffusion is two-dimensional in the dendrite and one-dimensional in the spine. C. Experimental Design: The role of anchoring is evaluated using four spatial variations in the location of adenylate cyclase and PKA. The adenylate cyclase-D1R complex (AC) is located either in the spine head or a focal dendritic submembrane area. Similarly, the PKA holoenzyme is located either in the spine head or the focal dendritic submembrane area. AMPA receptors containing GluA1 subunits are in the PSD compartment of the spine head for all cases.

The biochemical signaling network of direct pathway MSPNs (Fig. 1A, Table 1) was adapted from [27]. G_αolf_ coupled D1 type receptors connect dopamine stimulation to activation of adenylate cyclase [30], [31], with type 5 being the major type in the striatum [32]. cAMP produced by adenylate cyclase binds to the PKA holoenzyme, causing the two inherently active, catalytic subunits (PKAc) [33] to dissociate and diffuse away from the regulatory subunit dimer (PKAr). Alternatively, the cAMP saturated holoenzyme can bind to targets, which stabilizes dissociation of the catalytic subunits [34]. Targets of PKA include Thr34 of DARPP-32, Ser845 of the AMPA receptor GluA1 subunit [35], and phosphodiesterase type 10, which is the major phosphodiesterase in MSPN [36], [37]. Similar to the effect of PKA phosphorylation on phosphodiesterase type 4 [38], the activity of phosphodiesterase type 10 is enhanced by PKA phosphorylation. PhosphoThr34 DARPP-32 binds to and inhibits protein phosphatase 1 (PP1) [39]. DARPP-32 also can be phosphorylated on Thr75 by cdk5, and this form of DARPP-32 inhibits PKA [40].

**Table 1:** Reactions and rate constants of signaling pathways in the model.

Reaction Equation	kf (nM^−1^ s^−1^)	kb (s^−1^)	kcat (s^−1^)	Ref
Da⇌Da_Ext	2	2.00E-05		Adj
Da+R⇌DaR	0.00111	10		[27]
DaR+G_αβγ_⇌DaRG_αβγ_⇀DaRG_βγ_+G_α_GTP	6.00E-04	0.001	20	[27]
G_αβγ_+R⇌G_αβγ_R	6.00E-05	0.0003		[27]
G_αβγ_R+Da⇌DaRG_αβγ_⇀DaRG_βγ_+G_α_GTP	0.00333	10	20	PMR
DaRG_βγ_⇀DaR+G_βγ_	80			#
G_α_GTP⇀GaGDP	10			[27]
G_α_GDP+G_βγ_⇀G_αβγ_	100			[27]
G_αβγ_GTP+AC⇌ACG_α_GTP	0.0385	50		[27]
ACG_α_GTP+ATP⇌ACG_α_GTP−ATP	0.000128	0.2612		[27]
ACG_α_GTP−ATP⇌ACG_α_GTP+cAMP	28.46	0.000259		[27]
AC+Ca⇌ACCa	0.001	0.9		[27]
G_α_GTP+ACCa⇌ACG_α_GTPCa	0.01923	25		[27]
ACG_α_GTPCa+ATP⇌ACG_α_GTPCa−ATP	6.38E-05	0.1306		[27]
ACG_α_GTPCa−ATP⇌cAMP+ACG_α_GTPCa	14.23	0.00013		[27]
PDE1+CamCa_4_⇌PDE1CamCa_4_	0.1	1		[27]
PDE1CamCa_4_+cAMP⇌PDE1CamCa_4_cAMP⇀PDE1CamCa_4_+AMP	0.0046	44	11	[27]
PKAc+PDE10⇌PKAcPDE10⇀pPDE10+PKAc	6.25E-04	0.6022	0.1506	adj
PKAcAMP_4_+PDE10⇌PKAcAMP_4_PDE10	6.25E-05	0.6022		*
PKAcAMP_4_PDE10⇌PKAcPDE10+R2C_cAMP_4_	0.38	0.016		PMR
PKAc+PDE10cAMP⇌PKAcPDE10cAMP⇀pPDE10cAMP+PKAc	6.25E-04	0.6022	0.1506	adj
PKAcAMP_4_+PDE10cAMP⇌PKAcAMP_4_PDE10cAMP	6.25E-05	0.6022		*
PKAcAMP_4_PDE10cAMP⇌PKAcPDE10cAMP+R2C_cAMP_4_	0.38	0.016		PMR
pPDE10⇀PDE10	0.01036			Adj
PDE10+cAMP⇌PDE10cAMP⇀PDE10+AMP	0.084	16.8	4.2	[36]
pPDE10+cAMP⇌pPDE10cAMP ⇀ pPDE10+AMP	0.1008	16.8	8.4	adj
AMP⇀ATP	1			adj
Ca+pmca⇌pmcaCa⇀pmca+CaOut	0.05	7	3.5	[53]
Ca+ncx⇌ncxCa⇀ncx+CaOut	0.0168	11.2	5.6	[52]
CaOut⇌Ca	0.0017			adj
Ca+Calbindin⇌CalbindinCa	0.028	19.6		[50]
Cam+Ca_2_⇌CamCa_2_	0.006	9.1		[27]
CamCa_2_+Ca_2_⇌CamCa_4_	0.1	1000		[27]
Cam+PP2B⇌PP2BCam	0.0046	0.0012		PMR
CamCa_2_+PP2B⇌PP2BCamCa_2_	0.046	0.0012		[44]
PP2BCam+Ca_2_⇌PP2BCamCa_2_	0.006	0.91		[27]
CamCa_4_+PP2B⇌PP2BCamCa_4_	0.046	0.0012		[44]
PP2BCamCa_2_+Ca_2_⇌PP2BCamCa_4_	0.1	1000		PMR
CamCa_4_+CaMKII⇌CaMKIICamCa_4_	0.01	3		[118]
CaMKIICamCa_4_+CaMKIICamCa_4_⇌Complex	0.0001	10		&
CaMKIIpCamCa_4_+CaMKIICamCa_4_⇌pComplex	0.0001	10		&
CaMKIIpCamCa_4_+Complex⇀CaMKIIpCamCa_4_+pComplex	0.0001			&
CaMKIICamCa_4_+Complex⇀CamKIICamCa_4_+pComplex	0.0001			&
Complex+Complex⇀Complex+pComplex	0.01			&
Complex+pComplex⇀pComplex+pComplex	0.03			&
CaMKIIpCamCa_4_⇌CamCa_4_+CaMKIIp	0.0008	0.01		[118]
CaMKIIp+PP1⇌CaMKIIpPP1⇀PP1+CaMKII	1.00E-05	0.085	0.025	[119]
PKA+cAMP_2_⇌PKAcAMP_2_	8.70E-05	0.02		[33]
PKAcAMP_2_+cAMP_2_⇌PKAcAMP_4_	1.15E-04	0.2		[33]
PKAcAMP_4_⇌R2C_cAMP_4_+PKAc	0.038	0.016		[33]
R2C_cAMP_4_⇌PKAr+PKAc	0.152	0.004		adj
DARPP32+PKAc⇌DARPP32-PKA⇀PKAc+p34-DARPP32	0.0027	8	2	[27]
p34-DARPP32+PKAcAMP_4_⇌p34-DARPP32−PKAcAMP_4_	0.00027	8		*
p34-DARPP32−PKAcAMP_4_⇌R2C_cAMP_4_+DARPP32-PKA	0.38	0.016		PMR
p34-DARPP32+PP1⇌p34-DARPP32−PP1	0.4	0.58		[27]
p34-DARPP32+PP2BCamCa_4_⇌p34-DARPP32−PP2BCamCa_4_⇀PP2BCamCa_4_+DARPP32	0.000179	2	0.5	[46]
p34-DARPP32-PP1+PP2BCamCa_4_⇌p34-DARPP32-PP1-PP2BCamCa4⇀PP1-PP2BCamCa4+p34-DARPP32	2.98E-05	0.333	0.0833	[46]
PP1-PP2BCamCa_4_⇀PP2BCamCa_4_+PP1	5			#
p34-DARPP32+PP2A_X⇌p34-DARPP32-PP2A_X⇀PP2A_X+DARPP32	0.00152	56	14	[46], [56]
p34-DARPP32-PP1+PP2A_X⇌p34-DARPP32-PP1-PP2A_X⇀PP1-PP2A_X+DARPP32	0.000253	9.33	2.33	[46], [56]
PP1-PP2A_X ⇀ PP2A_X+PP1	50			#
Cdk5+DARPP32⇌CDK5D32⇀p75-DARPP32+Cdk5	0.0045	40	10	adj
p75-DARPP32+PKAc⇌p75-DARPP32−PKAc	0.00037	1		[27]
PP2A_B56d+PKAc⇌PKAcPP2A_B56d⇀PKAc+pPP2A	0.0025	0.3	0.1	[54]
PP2A_B56d+PKAcAMP_4_⇌PKAcAMP_4_PP2A_B56d	0.00025	0.3		*
PKAcAMP_4_PP2A_B56d⇌R2C_cAMP_4_+PKAcPP2A_B56d	0.38	0.016		PMR
PP2Ap⇌PP2A_B56d	0.004			adj
PP2A_BPR72+Ca⇌CaPP2A	3.33E-05	0.1		[55]
p75-DARPP32+pPP2A⇌p75-DARPP32-pPP2A⇀DARPP32+pPP2A	0.03	336	84	[54]
p75-DARPP32+PP2A_X⇌p75-DARPP32-PP2A_X⇀DARPP32+PP2A_X	0.0046	168	42	[55], [56]
p75-DARPP32+CaPP2A⇌p75-DARPP32-CaPP2A⇀CaPP2A+DARPP32	0.03	336	84	[55]
GluA1+PKAc⇌GluA1-PKAc⇀pS845-GluA1+PKAc	0.00402	24	6	[120]
PKAcAMP_4_+GluA1⇌GluA1−PKAcAMP_4_	0.000402	24		*
GluA1−PKAcAMP_4_⇌R2C_cAMP_4_+GluA1−PKAc	0.38	0.016		PMR
GluA1+CaMKIICamCa_4_⇌GluA1-CaMKIICamCa_4_⇀pS831-GluA1+CaMKIICaMCa_4_	2.22E-05	1.6	0.4	[120]
GluA1+CaMKIIpCamCa_4_⇌GluA1-CaMKIIpCamCa_4_⇀pS831-GluA1+CaMKIIpCamCa_4_	2.78E-05	2	0.5	[120]
GluA1+CaMKIIp⇌GluA1-CaMKIIp⇀pS831-GluA1+CaMKIIp	2.22E-05	1.6	0.4	[120]
pS845-GluA1+PP1⇌pS845-GluA1-PP1⇀GluA1+PP1	0.000218	0.17	0.0425	[120]
pS845pS831-GluA1+PP1⇌pS845pS831-GluA1-PP1⇀pS831-GluA1+PP1	0.000219	0.35	0.0875	[120]
pS831-GluA1+PP1⇌pS831-GluA1-PP1⇀GluA1+PP1	0.000219	0.35	0.0875	[120]
pS845-GluA1+PP2BCamCa_4_⇌pS845-GluA1 -PP2B⇀GluA1+PP2BCamCa_4_	0.00201	8	2	[120]

Rates were either obtained from the biochemical measurements, adjusted (adj) to reproduce other data, such as basal phosphoThr75-DARPP-32, or constrained by the thermodynamic principle of microscopic reversibility (PMR). R2C_cAMP_4_ is comprised of 2 regulatory PKA subunits and a single catalytic subunit.

*The cAMP saturated PKA holoenzyme likely binds to various targets at a lower rate than binding by the catalytic subunit [34]. Two types of reactions were added because NeuroRD is restricted to first or second order reactions:

& CaMKII phosphorylation reactions involving “Complex” are required to produce the observed calcium sensitivity [45], and capture the probability that two calmodulin bound CaMKII subunits are adjacent in the holoenzyme;

# Rapid dissociation after enzyme reaction prevents accumulation of these intermediate forms. PP2A_X indicate either BPR72 or B56d regulatory subunit. Phosphorylation of Ser845 and Ser831 are independent of each other, thus only one of two possible reactions are listed. Similarly, dephosphorylation of Ser831 is independent of whether Ser845 is phosphorylated or not, and dephosphorylation of Ser845 by calcineurin is independent of Ser831 phosphorylation.

Calcium either binds to and inhibits adenylate cyclase [41], or binds to calmodulin, which has four calcium-binding sites: one pair of fast, high affinity sites, and one pair of slow, low affinity sites [42], [43]. Calcium-bound-calmodulin binds to and activates several molecules in the striatum, including phosphodiesterase 1B, calcineurin [44], and calcium-calmodulin-dependent protein kinase type II (CaMKII) [45]. Activated calcineurin dephosphorylates phosphoThr34 DARPP-32 [46]; whereas activated CaMKII phosphorylates Ser 831 of GluA1 [47]. PP1 dephosphorylates both phosphoSer845 GluA1 and phosphoSer831 GluA1, whereas calcineurin dephosphorylates only phosphoSer845 GluA1 [48]. In addition to these molecules, to accurately simulate the calcium dynamics caused by cortical stimulation, calcium binds to the endogenous buffer calbindin [49], [50] and is extruded through the low affinity sodium calcium exchanger [51], [52], or the high affinity plasma membrane calcium ATPase pump [53].

Protein phosphatase 2A (PP2A) is a constitutively active phosphatase comprised of three subunits: catalytic, structural, and regulatory. In the model, half of the PP2A has the B56δ regulatory subunit and thus its activity is enhanced by PKA phosphorylation [54]. The remainder of the PP2A has the B″/PR72 regulatory subunit, and thus its activity is enhanced by binding a single calcium ion [55]. PP2A dephosphorylates phosphoThr75 DARPP-32 and also phosphoThr34 DARPP-32 [56].

The diffusion constant for each diffusible molecule was estimated as previously [57] but decreased due to cytosolic viscosity [58] to produce a calcium gradient from spine to dendrite similar to that measured experimentally [59]. The diffusible molecules included cAMP, ATP, all forms of calmodulin, CaMKII, DARPP-32 and the catalytic subunit of PKA (Table 2). To simulate anchoring, the diffusion coefficient of anchored molecules was set to 0. The molecules were anchored in specific regions by initializing the concentration to zero in all but the anchored regions in the morphology (Table 3,4). The anchored molecules included the dopamine receptor, G protein, adenylate cyclase, PKA (holoenzyme and regulatory subunits), phosphodiesterase and AMPA receptors. G proteins were colocalized with both the D1 receptor and adenylate cyclase for all simulations due to their limited mobility in the membrane [60] and as suggested experimentally [30], [61]. Anchored molecules can interact with either diffusible or anchored molecules that are in the same subvolume (see Morphology). To evaluate the role of colocalization, D1R, G proteins, adenylate cyclase, and PKA were anchored either in the spine head, or in a small dendritic region which had a volume equal to that of the spine head. Note that in neurons receptors are not completely immobilized in the membrane [62]; in addition, pools of PKA are anchored in multiple regions within the neuron. Therefore, non-diffusion of anchored molecules in our model serves to enhance the effect of colocalization, which facilitates evaluation of distinct functions of anchoring.

**Table 2:** Diffusion constants for diffusible molecules in the model.

Molecule Name	Diffusion Constant (µm^2^/sec)
Ca	174.3
Calbindin	9.3
CalbindinCa	9.3
Da	150
ATP	74.7
AMP	85.5
cAMP	86.4
Cam	11
CamCa_2_	11
CamCa_4_	11
CaMKIICamCa_4_	3.6
pCaMKIICamCa_4_	3.6
CaMKII	3.6
pCaMKII	3.6
PKAc	8.1
DARPP32	10.6
DARPP32-PKAc	10.6
pT34-DARPP32	10.6
pT75-DARPP32	10.6
pT75-DARPP32-PKAc	10.6

Molecules not listed do not diffuse; thus, their diffusion constants are zero. To calculate diffusion coefficients as in [57] we used a cytosolic viscosity of 4.1 for small molecules and a cytosolic viscosity of ∼8.7 for proteins . These values yielded calcium gradients similar to those measured experimentally [59], and diffusion constants similar to those measured experimentally [50].

**Table 3:** Initial concentrations of non-anchored molecule species in the simulation.

Molecule	General Cytosol (nM)
Ca	58.7
CaOut	2005230.1
Calbindin	145508.3
CalbindinCa	14329.0
Da	10.0
Da_ext	1000000.4
ATP	1998350.8
cAMP	35.6
PDE1	3125.4
PDE1CamCa_4_	855.5
PDE10	800.6
PDE10cAMP	118.3
pPDE10	364.3
pPDE10cAMP	34.0
AMP	606.0
CamCa_2_	341.9
Cam	4921.5
PP2BCam	2359.2
PP2BCamCa_2_	1218.4
PP2BCamCa_4_	8.1
CaMKII	11318.4
CaMKIIpCamCa_4_	140.0
CaMKIIp	500.0
CaMKIIpPP1	242.7
CaMKIIpCamCa_4_PP1	8.1
PP1	3750.0
PKA	1000.0
PKAcAMP_2_	160.0
PKAcAMP_4_	25.0
PKAc	50.0
PKAr	25.0
PP2A_BPR72	800.0
PP2A_B56d	630.0
DARPP32	36400.0
p34-DARPP32-PP1	650.0
p34-DARPP32-PP1-PP2A_B56d	11.3
Cdk5	1088.6
CDK5-DARPP32	2057.7
p75-DARPP32	10370.9
p75-DARPP32-PP2A_BPR72	200.0
p75-DARPP32-PP2A_B56d	200.0
p75-DARPP32-CaPP2A	25.0
p75-DARPP32-pPP2A	120.0
pPP2A	140.0
CaPP2A	33.0

Molecule	Dendrite Submembrane (picoSD)*
pmca	86.8
pmcaCa	28.3
ncx	2994.2
ncxCa	213.7
PDE10	292.5
PDE10cAMP	49.2
pPDE10	49.8
pPDE10cAMP	10.9

Molecule	Spine Cytosol (nM)#
pmca	297.8
pmcaCa	96.9
ncx	10268.5
ncxCa	733.0
PDE10	2500.0
PDE10cAMP	150.0
pPDE10	550.0
pPDE10cAMP	100.0

Molecule	PSD (nM)
GluA1	8050.7
pS845-GluA1	244.0
pS831-GluA1	853.9
pS845-GluA1-PP1	365.9

Molecules not listed have initial concentrations of 0. A single molecule produces a concentration of 28 nM in the dendrite subvolumes of the morphology; thus molecule concentrations less than 28 nM indicate that some subvolumes contained a single molecule and some did not, to produce the indicated concentration averaged over the entire morphology. General cytosol means that molecules populated the entire morphology.

*Molecules initialized in the dendrite submembrane are specified in picoMoles per µm^2^ (picoSD).

# Molecules initialized in the spine cytosol were excluded from the PSD.

**Table 4:** Initial concentrations of anchored molecule species in the simulation.

Anchored Molecules	Spine Cytosol (nM)#	Focal Dendrite Submembrane (picoSD)*
R	153.9	20.3
G	8158.3	1012.5
GR	1255.5	160.8
G_α_GTP	8.1	0.0
G_α_GDP	90.0	12.0
G_βγ_	43.2	0.0
AC	7428.0	887.6
ACG_α_GTPCa-ATP	14.0	0.0
ACG_α_GTP-ATP	114.5	19.0
ACCa	2376.0	259.9
PKA	7050.0	1130.0
PKAcAMP_2_	925.0	155.0
PKAcAMP_4_	137.5	30.0
PKAc	300.0	60.0
PKAr	150.0	30.0

Only one of these concentrations applied, depending on whether molecules were anchored in the spine, or in the dendrite.

*Molecules initialized in the dendrite submembrane are specified in picoMoles per µm^2^ (picoSD).

# Molecules initialized in the spine cytosol were excluded from the PSD, except for PKA species.

### Morphology

The biochemical network was simulated in a multi-compartment morphology comprised of a 7.75 µm long segment of dendrite (0.6 µm wide by 0.4 µm depth) with four spines (Fig. 1B). Each spine was comprised of a spine head (0.6 µm diameter), a neck (0.2 µm diameter and 0.3 µm long) and a post-synaptic density (PSD) [63]. The morphology was subdivided into multiple compartments in order to simulate the reactions and diffusion mesoscopically. The dendrite was subdivided into 310 cuboidal subvolumes of dimension 0.12×0.125×0.4 µm^3^, allowing 2-dimensional diffusion. One layer of dendritic subvolumes on either edge was considered as the submembrane region. The approximation of a cylindrical dendrite as a rectangular cuboid with a 0.12 µm width submembrane region produced the same ratio of submembrane to cytosol volume as a cylinder with a 70 nm submembrane region. Each spine was subdivided into 0.1 µm cylindrical slices, yielding 3 spine neck subvolumes, 2 spine head subvolumes and 1 PSD subvolume, permitting 1-dimensional diffusion. One set of simulations addressed the formation of microdomains using a 23 µm long dendrite with 12 spines. For these simulations, no molecules were anchored in the dendrite; thus it was subdivided into 342 subvolumes of dimension 0.2×0.2×0.4 µm ^3^ to improve simulation speed. Empirically, these subvolume sizes were both large enough to meet the well-stirred criterion [64] and smaller than the length scale of observed concentration gradients.

### Stimulation

The dopamine D1 receptors in the model were activated by 1 sec duration, 1 µM dopamine elevations similar to those measured using voltammetry during learning tasks in behaving animals [65]. This dopamine elevation was repeated 5 times with a 20 sec interval, similar to the intertrial interval used in behavioral tasks. In most simulations, the diffusion coefficient of dopamine was sufficiently fast to prevent any gradients, as proposed by the volume transmission hypothesis. In a subset of simulations, the diffusion coefficient was decreased to produce a small gradient in dopamine concentration, as suggested experimentally [66]. During one set of simulations dopamine and calcium were applied together. In these simulations, 100 pulses of calcium influx to the PSD region of two of the spines (3 msec duration, 10 msec inter pulse interval, 125 molecules/msec influx) approximates the calcium influx through NMDA receptors during 100 Hz, 1 sec stimulation [59] used for LTP protocols.

### Numerical Methods

NeuroRD [57] is used for computationally efficient, Monte Carlo (stochastic) simulation of reaction and diffusion of these signaling pathways. The stochastic algorithm is essential because many of the molecular populations are small, especially in the spine, invalidating the assumption of continuous concentration of molecules. Furthermore, the computational properties (i.e. bistability, threshold) of synaptic signaling pathways that are exhibited in deterministic models of spines are not necessarily maintained in stochastic models of spines [67]. NeuroRD is used because the total number of molecules in the morphology described (Fig. 1B, Table 3,4) is large enough to make simulation of individual molecules in microscopic stochastic simulators computationally prohibitive. NeuroRD achieves its efficiency by integrating the tau-leap stochastic reaction algorithm [68] with a computationally-efficient, tau-leap style of stochastic diffusion algorithm [69]. The leaping approach maintains accuracy while dramatically reducing the number of time-steps required for a simulation [70] as compared to spatial extensions of the Gillespie exact stochastic simulation algorithm [71]. Both the simulation software and the files used for the model simulations are freely available from modelDB (http://senselab.med.yale.edu/ModelDB/)and the authors website (http://krasnow.gmu.edu/CENlab/). Even with this accelerated algorithm, 500 sec of simulation time (using a simulation time step of 5 µsec) required 4 days for the 7.75 µm long dendrite and 7 days for the 23 µm long dendrite. Simulations in the 7.75 µm long dendrite were repeated 4 times using a different random number seed and simulations in the 23 µm long dendrite were repeated 6 times, analogous to repeated experimental trials. Numbers of molecules were converted into concentration, either in particular microdomains or in the entire morphology by dividing by the appropriate volume, to facilitate comparison between microdomains of different sizes. Because GluA1 is limited to the PSD, concentration is less meaningful; thus, the percent of receptors phosphorylated on Ser845 (both phosphorylated and unphosphorylated on Ser831) is reported.

## Results

Previous results have shown that PKA anchoring is important for striatal LTP [26] and striatal dependent behavior [72], but these experiments did not delineate whether PKA needs to be anchored near its target molecules, such as DARPP-32 and the AMPA receptor GluA1 subunit, or near a source of activator molecules, such as adenylate cyclase. Though disruption of PKA anchoring prevents phosphorylation of targets such as sodium channels [23] or adenylate cyclase 5 [73] in response to a general elevation in cAMP, the role of anchoring in response to spatially constrained cAMP has not been investigated. In order to clarify which of these two associations is critical to PKA function, a model of the signaling pathways implicated in striatal synaptic plasticity (Fig. 1A) was implemented using *NeuroRD* [57], in a 7.75 µm long dendrite with four spines (Fig. 1B).

### Validation of the Model

Prior to evaluating the role of PKA and adenylate cyclase location, the model was validated by comparison with experiments that measured phosphorylation of PKA targets in response to bath application of agonists. For this validation, both PKA and adenylate cyclase were located in the spine and dendrite in equal amounts.

**Figure 2:**
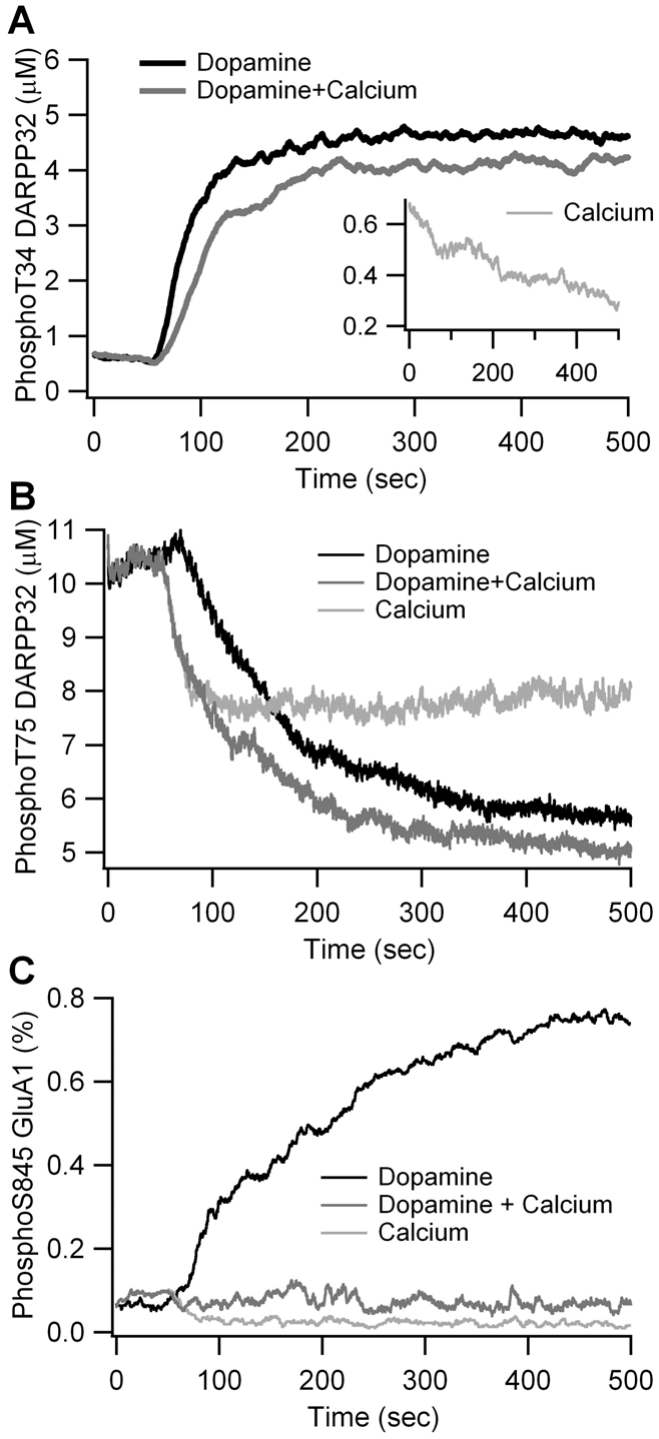
Validation of the model via simulation of agonist bath application. (A) Change in phosphoThr34 DARPP-32 is similar to that observed experimentally in response to 10 µM dopamine alone, 600 nM calcium alone (inset), and the combination of dopamine with calcium. (B) Decrease in phosphoThr75 DARPP-32 for same three conditions as (A). (C) Change in phosphoSer845 GluA1 for same three conditions as (A). All responses are similar to experimental measurements.

The first validation simulates the response to bath application of 10 µM dopamine. Fig. 2A shows that a simulated increase in dopamine from 10 nM (basal level) to 10 µM leads to a 6-fold increase in phosphoThr34 DARPP-32 within 2 min, and a decrease of phosphoThr75 DARPP-32 to 60% of basal (Fig. 2B) comparable to the changes measured experimentally using dopamine D1R agonists [74]. In addition, simulated phosphoSer845 GluA1 increases by 8 fold within 5 to 10 minutes (Fig. 2C), comparable to measurements performed in the absence of D2 receptor stimulation [48].

The second validation simulates the response to a sustained increase in intracellular calcium concentration, as occurs due to experimental activation of NMDA receptors and voltage dependent calcium channels [75]. In response to this calcium increase, simulated phosphorylation of both DARPP-32 residues decreases, to half (Thr34) or two-thirds (Thr75) the basal level (Fig. 2A and 2B), in agreement with experimental data [56], [76]. Furthermore, the simulated calcium elevation leads to dephosphorylation of phosphoSer845 GluA1 (Fig. 2C) as previously reported [75].

The last model validation evaluates the change in phosphorylation of Thr34 and Thr75 in response to paired dopamine and calcium elevations. As demonstrated experimentally [75], [76], the addition of calcium reduces the increase in phosphoThr34 caused by dopamine (Fig. 2A), and enhances the decrease in phosphoThr75 (Fig. 2B). Furthermore, paired elevation of dopamine and calcium completely blocks the simulated phosphorylation of GluA1 Ser845 (Fig. 2C).

### PKA Activity is Greater when Anchored near Adenylate Cyclase

Using this validated model, we explore the function of PKA anchoring relative to the location of both adenylate cyclase that activates it and PKA targets such as AMPA receptors (Fig. 1C). The D1 receptor is a metabotropic G protein coupled receptor functionally associated with adenylate cyclase [30], [31]. Immunohistochemical studies have shown D1 receptors located in spine heads and necks [77]. On the other hand, studies using immunogold labeling suggest D1 receptors are localized in extra synaptic sites homogeneously distributed in dendritic shafts and spines [78]. Therefore, prior to evaluating the effect of colocalization, we first examine the cAMP distribution when the adenylate cyclase/G protein/D1 receptor complex is located either in the spine head or in the submembrane region of the dendritic shaft.

**Figure 3:**
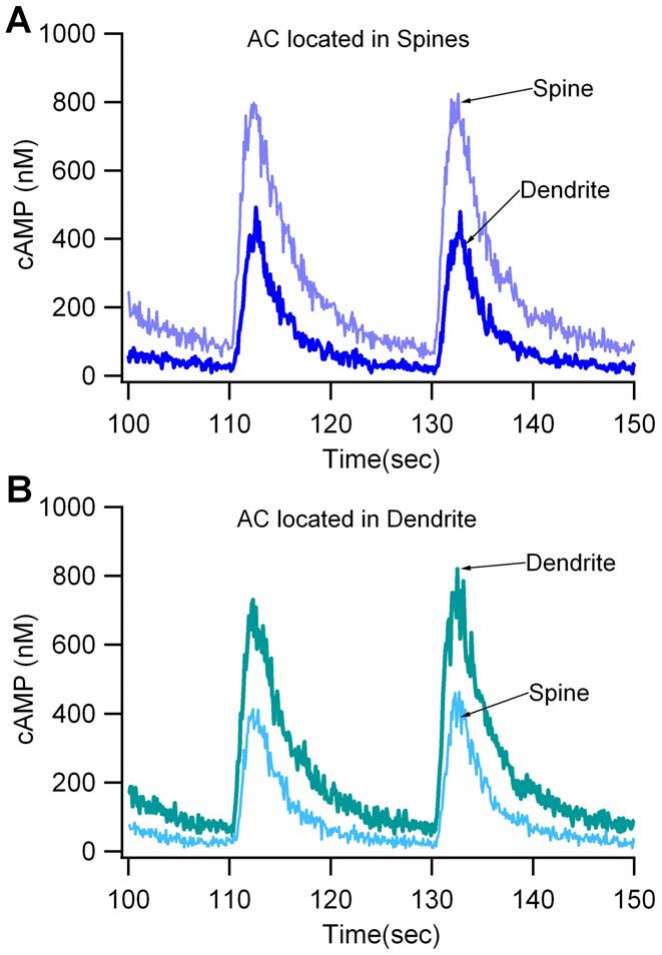
The gradient in cAMP concentration depends on the location of adenylate cyclase. (A) When adenylate cyclase (AC) is in the spine head, there is a large difference (gradient) between spine cAMP and dendrite cAMP. (B) When adenylate cyclase is in the dendrite, there is a small gradient from dendrite to spine.

Simulations show that when the adenylate cyclase-D1 receptor complex is located in the spine head, cAMP concentration is significantly higher in the spine head compared to the dendrite (Fig. 3A). This gradient is independent of PDE10 distribution (Suppl Fig S1). Conversely, when the complex is located in the submembrane compartment of the dendrite, cAMP concentration is higher in the dendrite than in the spine head (Fig. 3B). In summary, regardless of adenylate cyclase location, a cAMP gradient develops, with the highest concentration near the source (adenylate cyclase-D1 receptor complex); therefore, subsequent evaluations of the role of colocalization take into account the local cAMP concentration.

PKA location is regulated by interaction with A-Kinase anchoring proteins [19]. Various AKAPs anchor PKA to different locations, such as to the spine (e.g. AKAP5) [79], [80] or the dendritic shaft (e.g. MAP2) [81]. Recent evidence suggests that AKAP5 also anchors adenylate cyclase [22], [24], which would colocalize adenylate cyclase, PKA and GluA1 receptors. Alternatively, a pool of adenylate cyclase may be in the dendrite together with the dopamine D1 receptors found there [78]. In order to evaluate whether the critical function of anchoring is to place PKA near adenylate cyclase or near phosphoprotein targets, PKA is localized either in the spine heads or in a small submembrane region of the dendrite. For both of these spatial variations of PKA, the adenylate cyclase/D1 receptor complex is placed either in the spine heads or in the small submembrane region of the dendrite. Thus, four different spatial configurations are simulated (Fig. 1C). Note that the volume of the small dendrite region equals the volume of the spine head; thus, both global and local concentrations of these molecules are equal for all simulations. This 2×2 experimental design (Fig. 1C) allows assessment of the role of PKA location relative to adenylate cyclase - the source of cAMP, or a non-diffusible target - the AMPA receptor GluA1 subunit. Note that membrane associated molecules have limited mobility in the membrane [62] and pools of PKA are anchored in multiple regions within the neuron; nonetheless, our approach serves to enhance the effect of colocalization and facilitates evaluation of distinct functions of anchoring.

**Figure 4:**
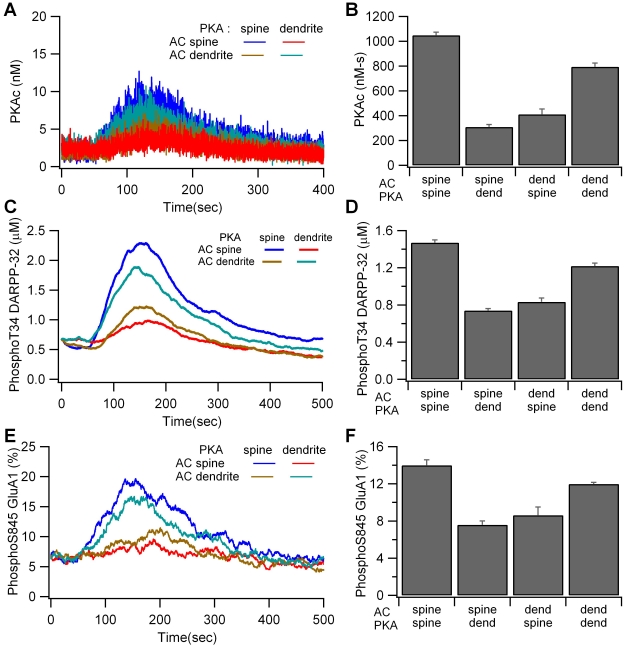
Colocalization of PKA with adenylate cyclase enhances PKA activity. (A, B) Concentration of free catalytic subunit (PKAc) is greater for the two colocalized cases. (A) Traces are averaged over 4 trials; nonetheless the stochastic fluctuations are so large that the traces overlay each other and are difficult to distinguish. (B) Mean and S.E.M. of the total PKA activity (PKAc summed between 50 and 350 s). (C,D) Concentration of phosphoThr34 DARPP-32 is greater for the two colocalized cases. (C) Traces are the average over four trials. (D) Mean and S.E.M. of the concentration of phosphoThr34 DARPP-32 averaged between 50 and 350 s. (E, F) Percent of phosphoSer845 GluA1 is greater for the two colocalized cases. (E) Traces are the average over four trials. (F) Mean and S.E.M. of the percent of phosphoSer845 GluA1 averaged between 50 and 300 s.

Simulations show that PKA colocalization with adenylate cyclase, either in the dendrite or the spine, produces a higher quantity of the active catalytic subunit than when PKA is apart from the adenylate cyclase (Fig. 4A, B). The large fluctuations in catalytic subunit are caused by its low quantity, which is due to a high affinity for the regulatory subunit and DARPP-32. Both the large fluctuations and the slow dynamics of PKA activation obscure the individual pulses that are visible in the cAMP traces. Colocalization in the spine produces greater PKA activity than colocalization in the dendrite because local spine cAMP is greater when adenylate cyclase is in the spine due to impeded cAMP diffusion. The effect of local cAMP (measured at the site of PKA anchoring) and colocalization (whether PKA was colocalized with adenylate cyclase or not) were evaluated using the SAS procedure GLM. Analysis revealed that local cAMP concentration predicted PKA activity (P<0.0001, R^2^ = 0.91), whereas colocalization did not reach significance (P = 0.083); thus the role of colocalization is primarily to place PKA in a microdomain of high cAMP concentration.

The effect of colocalization is propagated to downstream targets: Colocalization of PKA and adenylate cyclase (in spine or dendrite) leads to increased phosphoThr34 DARPP-32 (Fig. 4C,D) and phosphoSer845 GluA1 (Fig. 4E,F) compared to non-colocalized cases. In fact, both phosphoThr34 DARPP-32 and phosphoSer845 GluA1 are completely predicted by PKA activity (P = 0.0001, R^2^ = 0.999 and P = 0.0001, R^2^ = 0.93, respectively). The concentration of phosphoThr34 DARPP-32 (total quantity divided by volume of entire morphology) is illustrated because the gradients are much smaller than those of cAMP (Suppl Fig. S1). Evaluation of GluA1 in single spines on individual trials (Suppl Fig. S2) reveals that GluA1 phosphorylation varies considerably, exceeding 20% more often when PKA and adenylate cyclase are colocalized in the spine. This variability would not be evident in deterministic simulations.

**Figure 5:**
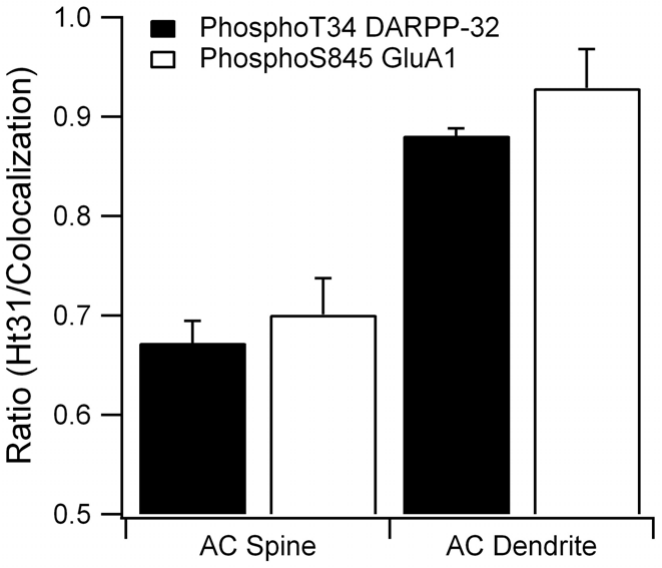
Disruption of PKA anchoring, as caused by Ht31 peptide, decreases PKA phosphorylation of downstream targets. When adenylate cyclase (AC) is in the spine, disruption of PKA anchoring reduces by 30% both phosphoThr34 DARPP-32 (p = 0.0006) and phosphoSer845 GluA1 (p = 0.0036). When adenylate cyclase is in the dendrite, disruption of PKA anchoring produces a significant decrease in phosphoThr34 DARPP-32 (p = 0.0005), but not phosphoSer845 GluA1 (p = 0.16). Most of the diffusely distributed PKA is in the dendrite; thus, cAMP diffusion out of the spine (when adenylate cyclase is in the spine) to reach the PKA is more difficult than cAMP diffusion within the dendrite (when adenylate cyclase is in the dendrite). Consequently, disruption of PKA anchoring has a larger effect when adenylate cyclase is in the spine. PhosphoThr34 DARPP-32 is averaged between 50 and 350 s and phosphoSer845 GluA1 is averaged between 50 and 300 s.

Previous experiments have shown that disruption of PKA anchoring using Ht31 peptide blocks L-LTP induction in hippocampus [25] and LTP induction in the striatum [26]. To evaluate this experimental observation, we performed simulations with PKA uniformly distributed in the morphology, mimicking the disruption of anchoring by Ht31 peptide [23], [25]. The simulation shows that phosphoThr34 DARPP-32 and phosphoSer845 GluA1 are reduced (ratio<1) when PKA is uniformly distributed (Fig. 5), especially for the case of adenylate cyclase located in the spine. This decreased PKA activity supports experimental studies showing that PKA anchoring is required for LTP induction.

### Spine Neck Length Enhances the Effect of Colocalization

Recent studies have demonstrated that synaptic function and plasticity are correlated with synaptic structure and spine morphology [82], [83]. In particular, spine-neck geometry is an important determinant of NMDA receptor-dependent calcium signaling in the dendrite [84], [85]. To evaluate how spine morphology affects the interaction between PKA and cAMP, simulations are repeated using a spine with either a longer (1.0 µm) or shorter (0 µm) spine neck, representing the range of experimentally measured values in striatal dendrites [86]. Keeping the spine head the same size and shape maintains both the same quantity and concentration of molecules localized in the spine head. For these simulations, the four configurations of PKA and adenylate cyclase introduced previously are used.

**Figure 6:**
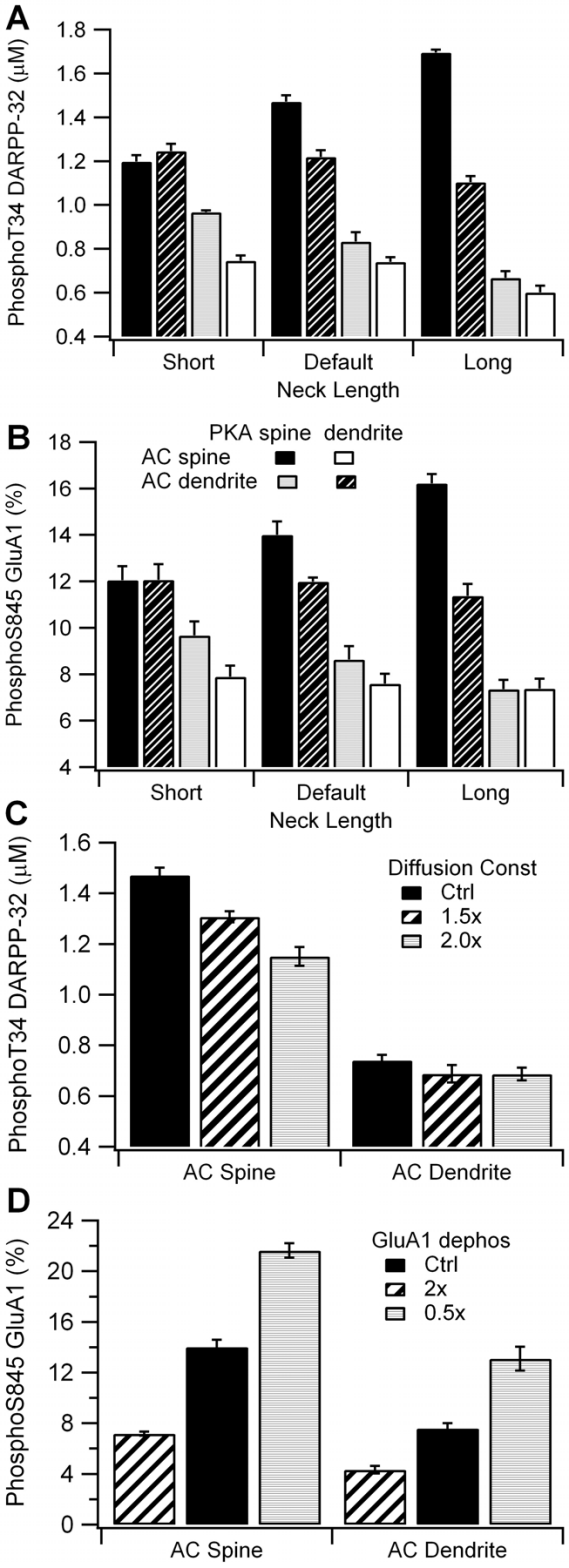
Results are robust to variation in parameters, though spine neck length enhances the effect of colocalization. (A) The difference in phosphoThr DARPP-32 between colocalized and non-colocalized cases increases with a longer spine neck. (B) The difference in phosphoSer845 GluA1 between colocalized and non-colocalized cases increases with spine neck length. (C) Increase in diffusion constant decreases the activity of PKA when colocalized in the spine with adenylate cyclase (AC), but maintains enhancement over the non-colocalized case. (D) Changes in rate of GluA1 phosphorylation changes the level of phosphoSer845 GluA1, but maintains the difference between colocalized and non-colocalized cases. PhosphoThr34 DARPP-32 is averaged between 50 and 350 s and phosphoSer845 GluA1 is averaged between 50 and 300 s.

Simulations show that spine neck enhances the effect of colocalization through control of cAMP concentration. An increase in spine neck length increases the cAMP gradient due to reduced diffusional coupling. Thus, an increase in neck length produces a larger cAMP in the spine when adenylate cyclase is in the spine, and a smaller cAMP in the spine when adenylate cyclase is in the dendrite. The effect of neck length on cAMP concentration gradient propagates downstream to PKA targets (Fig. 6A,B), yielding a larger effect of colocalization for the longer spine neck. Furthermore, local cAMP concentration predicts PKA activity regardless of neck length (P<0.0001, R^2^ = 0.93), and PKA activity predicts both phosphoThr34 DARPP-32 (P<0.0001, R^2^ = 0.99) and phosphoSer845 GluA1 (P<0.0001, R^2^ = 0.93). These results reinforce the observation that colocalization of PKA with its source of cAMP functions to locate PKA in a microdomain of high cAMP concentration, and also show that the effect of colocalization is robust to variation in spine neck length.

Several other simulations were performed to demonstrate that the results were robust to variations in parameters. The effect of colocalization was evaluated for different values of diffusion constants, rate of GluA1 dephosphorylation, and PDE10 location. For all these parameter variations, simulations demonstrate that colocalization of PKA with its activator still yields the greatest PKA activity. GluA1 dephosphorylation rate had no effect on PKA activity (results not shown), whereas an increase in diffusion constant (Fig. 6C) predictably decreased, but did not eliminate the effect of colocalization on PKA activity. Similarly, the GluA1 dephosphorylation rate changed the level of GluA1 Ser845 (Fig. 6D), but maintained the enhancement in phosphoSer845 GluA1 due to colocalization. Fig. S1 illustrates the effect of distributing PDE10 throughout the morphology (instead of locating it in the spine and submembrane region) for the two cases with adenylate cyclase in the spine. Though cAMP concentration and phosphoThr34 DARPP-32 are slightly increased when PKA is in the dendrite, PKA colocalization with adenylate cyclase still produces a much greater activity.

### Effect of Calcium on Dopamine Response

Experimental evidence demonstrates that calcium influx through NMDA receptors is required for plasticity [87], [88]; however the molecular mechanisms of calcium action are less clear. One possibility suggested by a previous model [27] is that *transient* calcium elevation (in contrast with prolonged calcium elevation) paired with dopamine enhances phosphoThr34 DARPP-32 compared with dopamine alone. Another possibility is that multiple kinases are required for synaptic plasticity, and, in particular, that calcium is required for CaMKII activation [45]. Therefore to explore the interaction between dopaminergic and glutamatergic transient signals in the medium spiny projection neuron, the model was stimulated with simultaneous dopamine and calcium pulses.

Transient calcium influx to spines was applied at 100 Hz for 1 sec, which approximates the calcium influx through NMDA receptors during LTP protocols [59]. This calcium influx generates a transient increase in calcium concentration that is constrained to the stimulated spines, as observed experimentally. Peak calcium levels in the stimulated spines (1500–2000 nM) are much higher than both the non-stimulated spines (500–600 nM) and the dendrite (∼400 nM), regardless of PKA and adenylate cyclase location.

**Figure 7:**
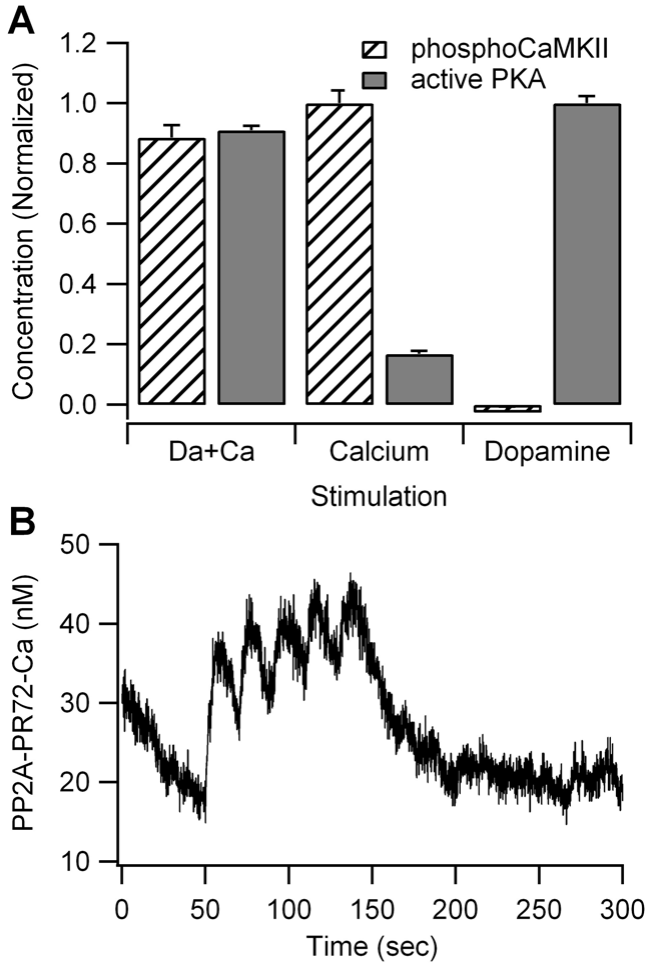
Effect of calcium on kinase activity. (A) Calcium alone produces a large increase in CaMKII phosphorylation and a small increase in PKA activity. Calcium together with dopamine leads to CaMKII phosphorylation only 10% lower than that observed with calcium alone, and PKA activity only 10% lower than that observed with dopamine alone. Both PKA and CaMKII activity are summed between 50 and 350 sec. (B) The calcium induced increase in PKA activity is caused by a small activation in PP2A, leading to a small decrease in phosphoThr75 (not shown).

Independent of whether PKA and adenylate cyclase were colocalized in the spine or dendrite, the PKA activity, phosphoThr34 DARPP-32, and phosphoSer845 were 10% smaller in response to calcium plus dopamine than in response to dopamine alone (Fig. 7). The inhibition in PKA activity is caused by three pathways: inhibition of adenylate cyclase 5, activation of phosphodiesterase 1B and activation of calcineurin that dephosphorylates phosphoThr34 DARPP-32. The small increase in PKA activity in response to calcium alone is due to enhanced dephosphorylation of phosphoThr75 DARPP-32 by calcium bound PP2A (Fig. 7B) which cannot compensate for the other inhibitory actions of calcium, regardless of PP2A distribution or affinity for calcium (results not shown).

To evaluate the possibility that calcium is needed for activation of other kinases, we measured the quantity of phosphorylated CaMKII, implicated in striatal LTP [89], in response to calcium, dopamine, and calcium plus dopamine. Fig. 7A shows that calcium stimulation alone causes a tremendous increase in phosphoCaMKII when compared with dopamine stimulation alone, while the combination of dopamine and calcium produces only a 10% reduction compared to calcium alone. This suggests that the increase in phosphoCaMKII more than compensates for the small decrease in PKA activity due to calcium. Though PKA and CaMKII have distinct targets in the model, extracellular signal-regulated kinase type II, important for dopamine signaling in the striatum [90], is phosphorylated by several kinases [91], and other points of PKA and CaMKII convergence are possible. In summary, these results suggest that both dopamine and calcium are required for LTP due to the need for activation of multiple kinases.

### Colocalization of Dopamine Terminals and Receptors

There are two conflicting hypotheses on the relationship between the extracellular concentration of dopamine and the activation of dopamine receptors [92]: either dopamine concentration exhibits a spatial gradient and preferentially activates receptors near terminals, or dopamine concentration does not exhibit a spatial gradient and activates receptors homogenously through volume transmission. The latter hypothesis is supported by the microscopic anatomy of dopamine receptors and terminals [78], [93]. On the other hand, voltammetry experiments coupled with modeling demonstrate a spatial gradient of dopamine concentration, suggesting that nearby low affinity receptors (e.g. D1R) are activated preferentially [66], [94], [95]. Therefore the next set of simulations evaluates the extent to which an extracellular microdomain in dopamine would produce an intracellular microdomain of D1R activated second messengers.

To approximate the spatio-temporal profile of dopamine observed in voltammetry experiments [66], dopamine diffusion was decreased by a factor of 3. This approach avoided the need for dopamine uptake via the dopamine transporter and subsequent degradation, which was beyond the scope of the current paper that focuses on post-synaptic mechanisms. The quantity of dopamine released at the spine was adjusted so that dopamine concentration at the release site remained the same, which yielded no change in PKA activity when PKA, D1R and adenylate cyclase were colocalized with the dopamine terminals in the spine. The effect of the resulting dopamine spatial gradient was evaluated by measuring the change in PKA activity when PKA, D1R and adenylate cyclase were colocalized in the dendrite, but 3 µm from the dopamine terminals.

**Figure 8:**
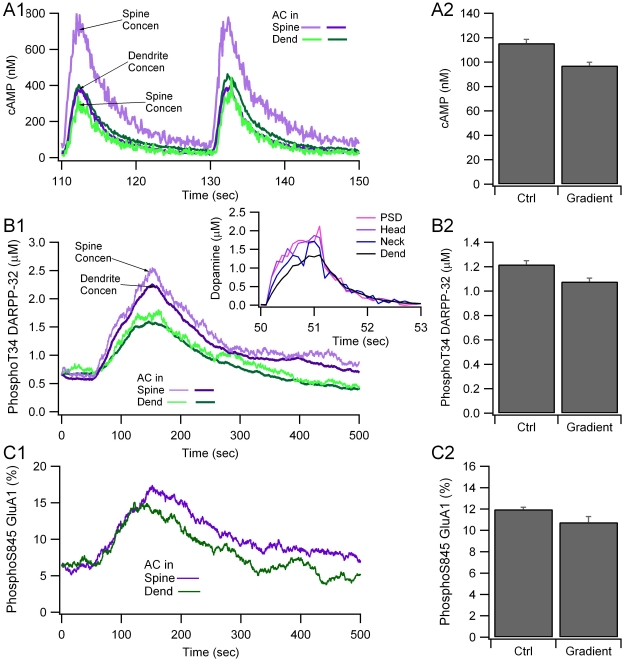
Locating dopamine receptors several microns away from the dopamine release site produces only a small change in cAMP signaling. A spatial gradient of dopamine decreases (A) cAMP and PKA activity as measured by (B) phosphoThr34 DARPP-32 and (C) phosphoSer845 GluA1, but no delay in time course. A1, B1 and C1 show the time course of a single simulation, A2 show mean and stdev (n = 4) of cAMP averaged between 50 and 200 s; B2 and C2 show mean and stdev (n = 4) of phosphoThr34 DARPP-32 averaged between 50 and 350 s, and phosphoSer845 GluA1 averaged between 50 and 300 s, respectively. The inset of B1 shows the gradient in dopamine concentration from the PSD to the dendrite. Traces are the average of four simulations.

Simulations show that the dopamine spatial gradient (Fig. 8B1 inset) leads to a small but significant decrease in amplitude of cAMP concentration (p = 0.0012) and phosphoThr34 DARPP-32 (p = 0.046), compared to no dopamine gradient (Fig. 8). In contrast, the time of the peak cAMP concentration (Fig. 8A1), phosphoThr34 DARPP-32 (Fig. 8B1) and phosphoSer845 GluA1 (Fig. 8C1) were not altered. This small change in intracellular signaling molecules caused by the dopamine spatial gradient suggests that small distances between receptor and terminal may not be important, especially if the intracellular molecules do not exhibit a spatial gradient. This idea was further evaluated with the next simulation, in which spatial gradients of intracellular signaling molecules were evaluated in a longer dendrite.

### Microdomains and Spatial Specificity

The effect of close proximity between dopamine terminals and receptors can only be important when accompanied by mechanisms for limiting the spread of intracellular signaling molecules. Spatial specificity of intracellular signaling is critical for information processing, in particular for a neuron to discriminate between different patterns of input. To evaluate if spatial specificity of dopamine signaling would propagate to downstream targets, we performed simulations using a longer dendrite with an average spine density of 1/µm, as experimentally measured [86]. These simulations use the slow dopamine diffusion which produces a gradient in dopamine to ensure that only the dopamine receptors at one end are activated.

**Figure 9:**
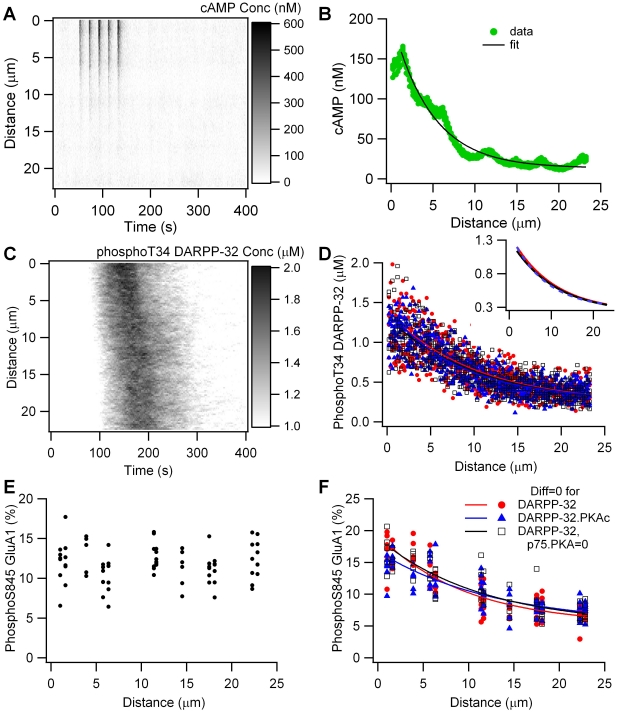
Dopamine gradients produce intracellular gradients of cAMP, but not PKA activity. (A) cAMP concentration versus time and distance from dopamine release site. (B) cAMP concentration, averaged from 50 to 150 sec, is well fit by single exponential decay. (C) phosphoThr34 DARPP-32 concentration versus time and distance from dopamine release site exhibits minimal spatial gradient. (D) Concentration of phosphoThr34 DARPP-32, averaged between 100 and 250 sec, exhibits a spatial gradient when diffusion of all DARPP-32 forms is zero (red), or diffusion of PKA bound DARPP-32 is zero (blue). Blocking the phosphoThr75-PKA interaction does not change the gradient that appears when diffusion of all DARPP-32 forms is zero (black). All three cases overlap and have the same decay space constant; thus, they are difficult to distinguish in the figure. The inset shows the fits alone, which also overlap. (E) Percent of GluA1 phosphorylated on Ser845, averaged between 100 and 250 sec, versus distance from dopamine release site. (F) Percent of GluA1 phosphorylated on Ser845, averaged between 100 and 250 sec, exhibits a spatial gradient when diffusion of the DARPP-32 forms is zero (red), or diffusion of PKA bound DARPP-32 is zero (blue). Blocking the phosphoThr75-PKA interaction does not change the gradient that appears when diffusion of all DARPP-32 forms is zero (black).

Fig. 9A shows that dopamine stimulation of one end of the dendrite produces a spatial gradient in cAMP concentration, reaching ∼400 nM at the stimulated end, and decaying to basal concentration within 13 µm of the stimulation site. The spatial profile of cAMP is well described by a single spatial decay constant of 4.69±0.14 µm (Fig. 9B). This gradient does not propagate to PKA activity, as measured by phosphoThr34 DARPP-32 activity (Fig. 9C), or phosphoSer845 GluA1 (Fig. 9E). The lack of intracellular gradient of PKA activity supports the ineffectiveness of extracellular gradients in dopamine. This result further suggests that PKA anchoring is not sufficient by itself to produce PKA microdomains, in part because when PKA is activated, the catalytic subunit diffuses.

The decay length of a molecule's concentration gradient is governed not only by its diffusion constant but also by its inactivation rate [17], [96]. Though PKA is inactivated by the large quantity (∼11 µM) of phosphoThr75 DARPP-32, this inactivation is reversible, similar to calcium binding to calcium buffers [97]. In addition, diffusion of phosphoThr34 DARPP-32 could obscure the gradient of PKA activity. Therefore, to evaluate the role of DARPP-32, simulations were repeated with diffusion constants for various forms of DARPP-32 set to zero.

Fig. 9D and 9F show that a gradient in phosphoThr34 DARPP-32 and phosphoSer845 GluA1 is observed in the absence of diffusion of any form of DARPP-32 (red traces). The gradient has a spatial decay constant of 14.7±8.5 µm for phosphoThr34 DARPP-32, which does not differ significantly from the spatial decay constant of 16.1±12.2 µm for phosphoSer845 GluA1 (P = 0.84). To demonstrate that DARPP-32 acts similar to calcium buffers in spreading PKA activity, simulations were repeated with diffusion for only the PKA-bound forms set to zero (both the PKA-DARPP-32 complex and the inhibited PKA-phosphoThr75 DARPP-32 complex), but diffusion of phosphoThr34 DARPP-32 enabled. Figs. 9D and 9E show that a gradient in phosphoThr34 DARPP-32 and phosphoSer845 GluA1 are observed in these conditions (blue traces). More importantly, the spatial decay constants of the gradient do not differ between these two cases (P = 0.8 for phosphoThr34 DARPP-32 and P = 0.4 for phosphoSer845 GluA1). As a further demonstration that PKA binding to phosphoThr75 DARPP-32 does not act as an inactivation mechanism, simulations were repeated with this reaction blocked, in the absence of DARPP-32 diffusion. Fig. 9D and 9F (black traces) show that blocking this reaction does not decrease the gradient, and thus the reaction does not act as an inactivation mechanism at this spatial scale.

## Discussion

The requirement of PKA activity for striatal synaptic plasticity in both D1R and D2R containing medium spiny projection neurons is well known [98], [99], but this research demonstrates that the subcellular location of PKA is critical, a prediction that has been confirmed experimentally [26]. AKAPs spatially constrain PKA through the organization of macromolecular complexes that effectively colocalize activators and effectors of enzymes. We investigated whether the critical function of AKAPs is to localize PKA near target proteins or near the source of cAMP, using a multi-compartmental stochastic reaction-diffusion model of the signaling pathways leading to PKA activation in medium spiny projection neurons. Simulations show that anchoring PKA near adenylate cyclase, the source of cAMP, is crucial because it places PKA in a high cAMP concentration microdomain. Such microdomains are created when inactivation, i.e., by phosphodiesterases, is strong enough to prevent the widespread elevation of cAMP elsewhere in the cell [18], [57]. Anchoring of PKA near its phosphoprotein targets is less important because PKA activity is less constrained due to the weak inactivation mechanisms and the spread of PKA bound to DARPP-32. This result leads to the experimental prediction that disruption of LTP by Ht31 peptide [24] will be rescued by phosphodiesterase inhibitors, which increase the diffusional distance of cAMP.

These simulations have behavioral implications because striatal dependent reward learning is associated with cortical glutamatergic stimulation followed by dopamine release in the striatum. One mechanism to ensure enhancement of only those synapses that assist in obtaining the reward is for dopamine to interact preferentially with a subset of synapses to create intracellular microdomains of cAMP. Though evidence suggests that dopamine is spatially unconstrained [78], [93], our simulations investigated whether an extracellular spatial gradient of dopamine could support an intracellular, spatial gradient of PKA activity. The results showed that the extracellular dopamine gradient produces a cAMP gradient, but not a gradient of phosphorylated PKA targets: neither phosphoThr34 DARPP-32 nor phosphoSer845 GluA1. Had the extracellular dopamine gradient been steeper, as in [94], the intracellular cAMP gradient may have been steeper. Nonetheless, the lack of inactivation mechanisms for PKA, and the diffusion of PKA bound forms of DARPP-32 would still minimize, if not eliminate, the gradient in PKA activity. On the other hand, though a gradient of PKA activity was not observed, the time of peak phosphoThr34 DARPP-32 was delayed in the part of the dendrite furthest from the dopamine release site. This result suggests that molecules sensitive to the temporal interval between PKA activity and calcium elevation could provide spatial specificity, and supports the hypothesis that dopamine provides synaptic specificity through temporal association with glutamatergic inputs [92].

The lack of gradient in PKA activity is due to weak inactivation mechanisms of PKA, which is surprising given the 11 µM concentration of phosphoT75-DARPP-32. It also seems inconsistent with prior demonstrations that disruption of PKA anchoring blocks phosphorylation of sodium channels [23] and calcium channels [100] in response to a general cAMP elevation. It is important to note that our results do not demonstrate that anchoring near targets is *un*important, just that anchoring near cyclase is *more* important in response to synaptic activation. Indeed, anchoring of PKA and adenylate cyclase in the spine produced greater phosphorylation of GluA1 than when PKA and adenylate cyclase were anchored in the dendrite. Enriching phosphatases in the PSD near the GluA1 receptor did not enhance the effect of colocalizing PKA with GluA1 (results not shown), suggesting the need to identify specific inactivation mechanisms for PKA to exhibit spatial specificity.

Neurons may have other inactivation mechanisms for PKA which are not included in our model. For example, multiple pools of anchored PKA coexist in a single neuron and distributing PKA throughout the neuron may have accelerated PKA inactivation. These distinct pools of anchored PKA probably phosphorylate unique post-synaptic effectors that are important for learning, addiction and synaptic plasticity. For example, PKA anchored in the spine may phosphorylate proteins in the spine head, of which GluA1 Ser845 is the example used in our simulations because it is upregulated in LTP [11]. Other pools of PKA, either in the dendrite or in the soma, may phosphorylate DARPP-32, CREB and molecules of the ERK pathway [101], which are implicated in synaptic plasticity [102] and the nuclear response to psychostimulants [103]. Nonetheless, the purpose of simulating the anchoring of PKA in a single location was to evaluate whether individual pools of PKA are spatially constrained.

Another possible PKA inactivation mechanism is excess quantity of regulatory subunit, as suggested in recent studies of cardiac myocytes [104], which might increase the importance of anchoring near targets and reveal a gradient even in the presence of DARPP-32 diffusion. A third possibility is a dynamic increased in phosphoThr75-DARPP-32 which requires modulation of cdk5 activity or location [105]. As more information about these signaling pathways becomes available, they can be evaluated by incorporation into the model. Alternatively, a gradient is likely to appear with focal dopamine release onto a much longer dendrite, given that a spatial gradient of PKA activity was observed in a model of hippocampal dendrites [106]. Nevertheless, the diffuse distribution of dopamine terminals makes it unlikely for a spatial gradient to develop under physiological conditions without stronger inactivation mechanisms. Ultimately, neuronal imaging using the AKAR sensor [107] of the spatial extent of PKA activity in response to focal application of dopamine is required to validate this model result.

Though inactivation mechanisms are clearly important for generation of spatial microdomains, morphology also plays an important role. Though qualitatively the results were robust to the length of the spine neck, quantitatively a longer spine neck enhanced the effect of colocalization, and a shorter spine neck decreased the effect of colocalization. This effect of spine neck may contribute to a positive feedback loop of synaptic regulation. A number of imaging studies have demonstrated that structural plasticity is correlated with functional synaptic plasticity [82]. In particular, LTP induction leads to longer spines [15], which would enhance the activation of PKA and possibly other molecules anchored in the spine head, such as Rap1GAP [108], which in turn would further enhance spine size.

The reduction in PKA activity caused by transient elevation in calcium contrasts with the results of previous models that show a calcium induced enhancement of either phosphoThr34 DARPP-32 [27] or phosphorylated AMPA receptors [29]. In the striatum, calcium inhibits PKA activity through its direct inhibition of adenylate cyclase 5, and calcium-calmodulin dependent activation of phosphodiesterase type 1B. The only mechanism for calcium enhancement of PKA activity in our model is calcium activation of protein phosphatase 2A, which enhances dephosphorylation of phosphoThr75 DARPP-32. In contrast to the Lindskog model, dependence of protein phosphatase 2A on calcium is first order, consistent with recent experiments [55], [109]. Therefore, the small amplitude of calcium bound PP2A (individual pulses clearly discernable in Fig. 7B) and inhibition of phosphoThr75 DARPP-32 is insufficient to compensate for other inhibitory actions of calcium.

This result is puzzling because experiments clearly demonstrate that both NMDA receptors and dopamine are required for synaptic plasticity [87], [88]. One possible post-synaptic action of calcium, supported by this model, is activation of CaMKII [89]; however, other calcium activated molecules, such as PKC [7], may be important. In addition, calcium activates calDAG GEF1 [110] and RasGRF [111] both of which lead to ERK activation, which modulates striatal LTP [102]. Another pathway is activation of casein kinase 1 by calcineurin, which then phosphorylates Ser137 of DARPP-32, thereby impairing the ability of calcineurin to dephosphorylate phosphoThr34 DARPP-32. Though this pathway was included in prior computational models [28], [29], it did not lead to synergistic phosphorylation of Thr34 DARPP-32. Nonetheless, colocalizing casein kinase 1 near calcineurin may produce such synergistic activation and should be explored in future studies. In addition to these interactions between calcium and dopamine pathways, direct interactions between dopamine and NMDA receptors may be important. Pre-synaptic glutamate receptors may modulate dopamine release [112], though NMDA receptors in dopamine neurons do not appear necessary for classical conditioning [113]. Alternatively, PKA phosphorylation of striatal enriched phosphatase (STEP) inactivates STEP, decreasing NMDA dephosphorylation and endocytosis [114].

Dynamic anchoring of several other molecules may be important for synaptic plasticity. CaMKII anchors at the PSD in an activity dependent manner, enhancing its ability to phosphorylate GluA1 receptors and protecting them from dephosphorylation [115]. Phosphodiesterase type 10 is primarily membrane associated, but moves to the cytosol when phosphorylated by PKA [116]. This translocation could accelerate degradation of cytosolic cAMP while allowing greater elevations in submembrane cAMP. Calcineurin also anchors to AKAP5 [117], which would enhance its activation by calcium in the spine, and make it less sensitive to calcium influx in the dendrite. The control of molecule anchoring by binding reactions or post-translational modification adds yet another mechanism for flexible regulation of signaling pathways.

## Supporting Information

Figure S1 Diffuse distribution of PDE10 does not change the effect of colocalization. (A) cAMP: diffuse distribution of PDE10 (A2) enhances the cAMP concentration compared to control (A1) when PKA is in the dendrite, but not when PKA is in the spine. There is no change in the spine to dendrite gradient. (B) phosphoT34 DARPP-32: diffuse distribution of PDE10 (B2) increases phosphoT34 DARPP-32 slightlycompared to control (B1) when PKA is in the dendrite, probably due to the increased cAMP concentration. (C) phosphoS845 Glur1: No effect of PDE10 location is observed.
(JPG)

Figure S2 Variability in the phosphorylation of S845 GluR1. (A) AC, PKA colocalized in the Dendrite; (B) AC, PKA colocalized in the spine. Top row shows results for one random seed; bottom row shows results for a different random seed. The percent phosphorylation, and the spine with greatest GluR1 phosphorylation differs between trials.
(JPG)
