# Supplementary figures and images for: Subcellular Location of PKA Controls Striatal Plasticity: Stochastic Simulations in Spiny Dendrites
Source: PLoS Comput Biol. 2012 Feb 9;8(2):e1002383. doi: 10.1371/journal.pcbi.1002383 (PMC3276550; doi:10.1371/journal.pcbi.1002383)

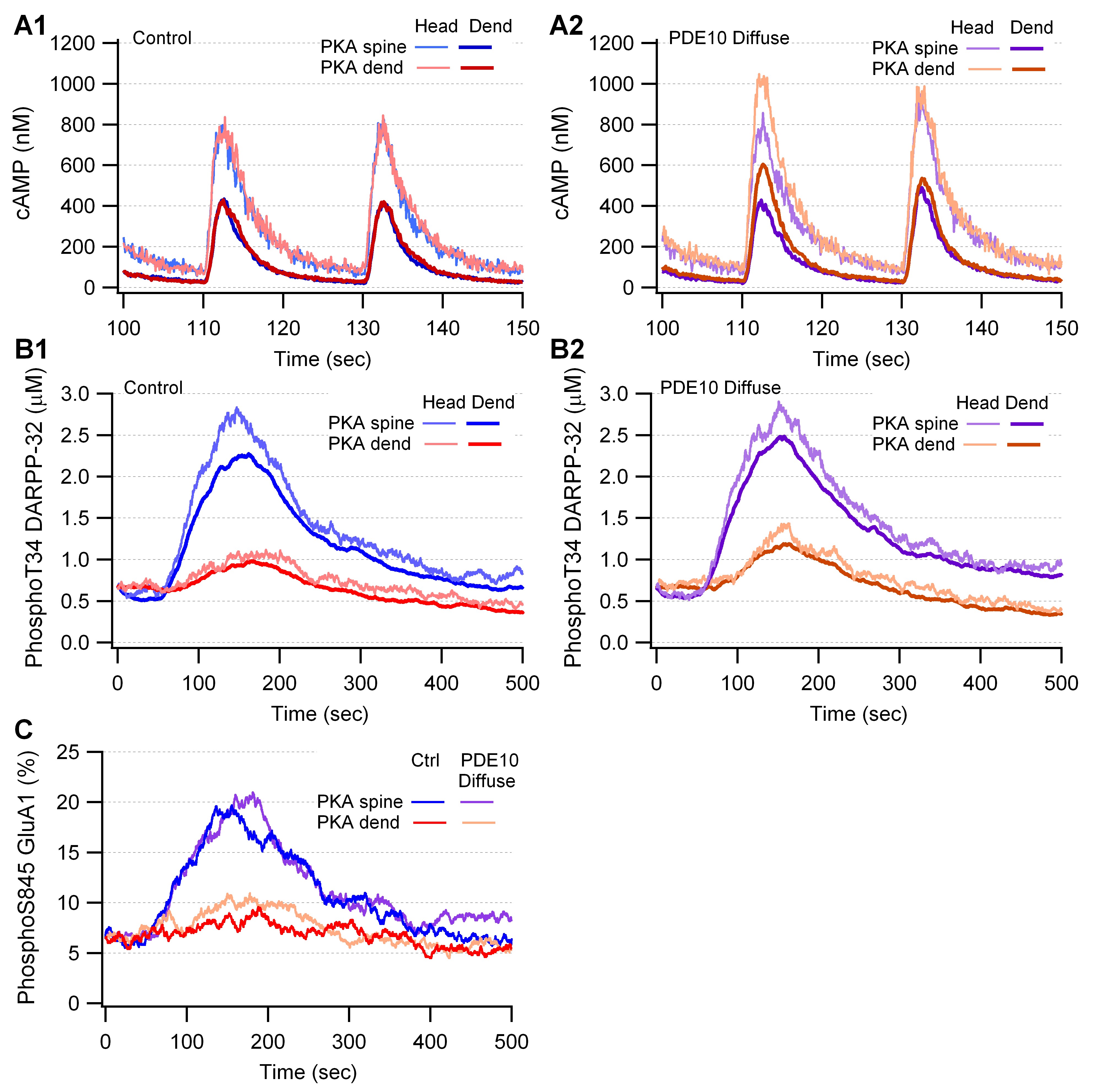

Supplement: Figure S1 — Diffuse distribution of PDE10 does not change the effect of colocalization. (A) cAMP: diffuse distribution of PDE10 (A2) enhances the cAMP concentration compared to control (A1) when PKA is in the dendrite, but not when PKA is in the spine. There is no change in the spine to dendrite gradient. (B) phosphoT34 DARPP-32: diffuse distribution of PDE10 (B2) increases phosphoT34 DARPP-32 slightlycompared to control (B1) when PKA is in the dendrite, probably due to the increased cAMP concentration. (C) phosphoS845 Glur1: No effect of PDE10 location is observed. (JPG) [file pcbi.1002383.s001.jpg]

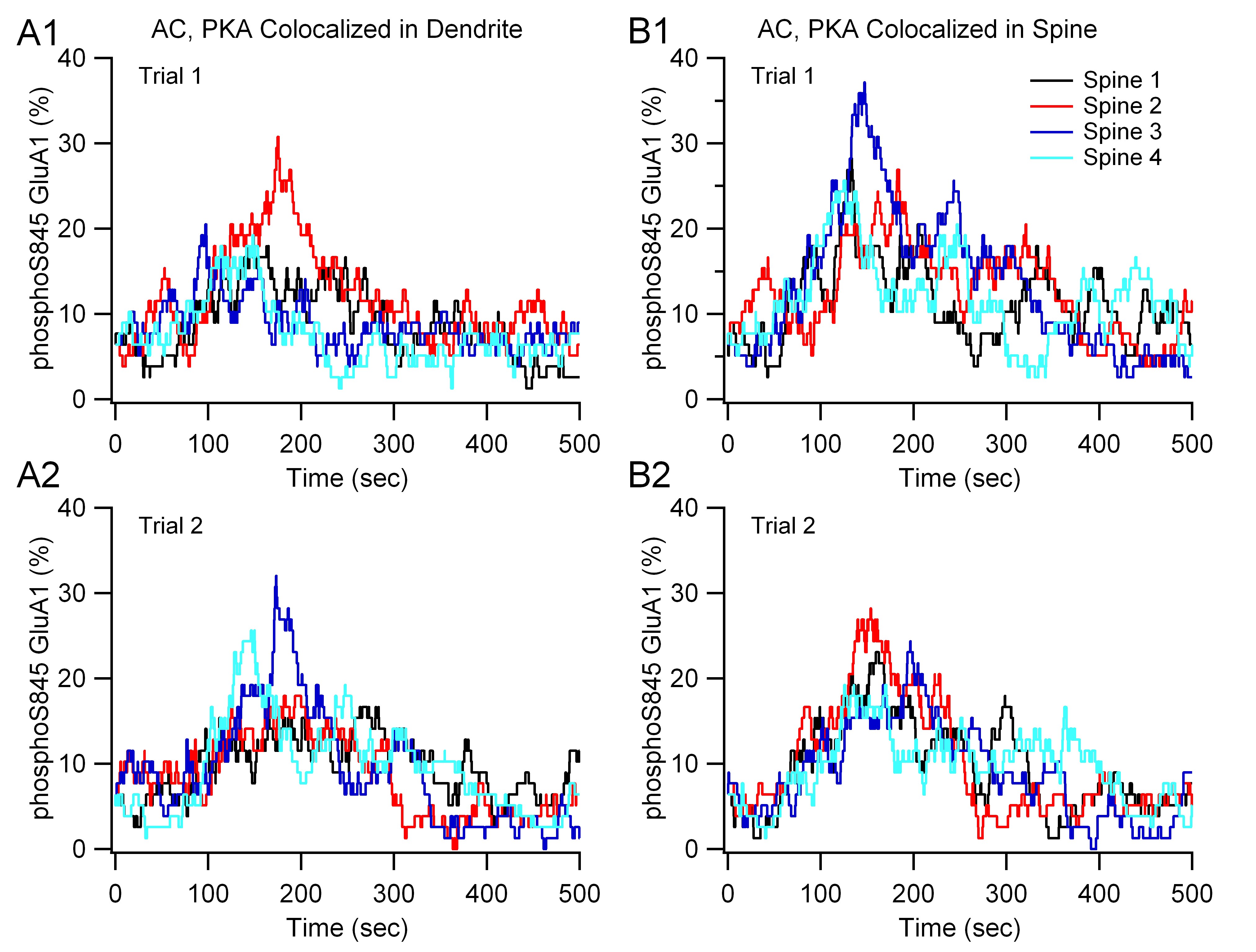

Supplement: Figure S2 — Variability in the phosphorylation of S845 GluR1. (A) AC, PKA colocalized in the Dendrite; (B) AC, PKA colocalized in the spine. Top row shows results for one random seed; bottom row shows results for a different random seed. The percent phosphorylation, and the spine with greatest GluR1 phosphorylation differs between trials. (JPG) [file pcbi.1002383.s002.jpg]
